# From policy to practice: implementing frontline community health services for substance dependence–study protocol

**DOI:** 10.1186/s13012-014-0108-x

**Published:** 2014-08-20

**Authors:** Kathryn J Gill, Emily Campbell, Gail Gauthier, Spyridoula Xenocostas, Dara Charney, Ann C Macaulay, other members of the CIHR Team in Substance Abuse Treatment

**Affiliations:** Addictions Unit, McGill University Health Centre, 1547 Pine Avenue West, Montreal, Quebec Canada; Psychiatry Department, McGill University, 1033 Pine Avenue West, Montreal, Quebec Canada; Centre de Santé et de Services Sociaux de la Montagne, 1801 Boulevard de Maisonneuve Ouest, 6e étage, Montréal, Québec Canada; Participatory Research at McGill, Department of Family Medicine, McGill University, 5858 Chemin de la Côte-des-Neiges, 3rd floor, Montreal, Quebec Canada

**Keywords:** Substance dependence, Screening, Brief interventions, Frontline health services, Implementation, Knowledge translation, Policy, Organizational change

## Abstract

**Background:**

Substance abuse is a worldwide public health concern. Extensive scientific research has shown that screening and brief interventions for substance use disorders administered in primary care provide substantial benefit at relatively low cost. Frontline health clinicians are well placed to detect and treat patients with substance use disorders. Despite effectiveness shown in research, there are many factors that impact the implementation of these practices in real-world clinical practice. Recently, the Ministry of Health and Social Services in Quebec, Canada, issued two policy documents aimed at introducing screening and early intervention for substance abuse into frontline healthcare clinics in Quebec. The current research protocol was developed in order to study the process of implementation of evidence-based addiction treatment practices at three primary care clinics in Montreal (Phase 1). In addition, the research protocol was designed to examine the efficacy of overall policy implementation, including barriers and facilitators to addictions program development throughout Quebec (Phase 2).

**Methods/Design:**

Phase 1 will provide an in-depth case study of knowledge translation and implementation. The study protocol will utilize an integrated knowledge translation strategy to build collaborative mechanisms for knowledge exchange between researchers, addiction specialists, and frontline practitioners (guided by the principles of participatory-action research), and directly examine the process of knowledge uptake and barriers to transfer using both qualitative and quantitative methodologies. Evaluation will involve multiple measures, time points and domains; program uptake and effectiveness will be determined by changes in healthcare service delivery, sustainability and outcomes. In Phase 2, qualitative methods will be utilized to examine the contextual facilitators and barriers that frontline organizations face in implementing services for substance dependence. Phase 2 will provide the first study exploring the wide-scale implementation of frontline services for substance dependence in the province of Quebec and yield needed information about how to effectively implement mandated policies into clinical practice and impact public health.

**Discussion:**

Findings from this research program will contribute to the understanding of factors associated with implementation of frontline services for substance dependence and help to inform future policy and organizational support for the implementation of evidence-based practices.

## Background

Alcohol and drug abusers are among the highest cost users of the healthcare system in North America. For example, the total annual healthcare costs of tobacco, alcohol and drugs in the United States is estimated at $137 billion, while the overall costs including health care, crime and lost work productivity is estimated at more than $600 billion [1]. The total economic costs of substance dependence in Canada alone have been estimated at approximately $40 billion annually [2]. Research has repeatedly demonstrated that effective treatment reduces health and social service demand by substance abusers and their family members [3–5]. Thus, effective and timely intervention at primary points of contact with the healthcare system could produce a significant reduction in the long term morbidity associated with untreated substance dependence^a^ such as HIV/AIDS, hepatitis B and C, tuberculosis and liver cirrhosis, as well as improvements in psychosocial functioning and quality of life. However, frontline practitioners often fall short in identification of problem alcohol and drug users, as well as in the delivery of appropriate interventions [6–8].

Extensive scientific research has shown that screening and brief interventions for substance use disorders administered in primary care provide substantial benefit at relatively low cost [9–13]. Brief interventions (BI) are typically defined as consisting of five (or fewer) sessions, and include an assessment of alcohol/drug intake, feedback on negative consequences, clear advice to reduce or abstain, motivational techniques to enhance self-efficacy, as well as manuals and information on self-help groups and other resources within the community. A large body of evidence now shows that BI tends to perform as well as more extended treatments over a wide range of outcomes [14]. Most recently, large scale international studies conducted by the World Health Organization demonstrated the effectiveness of BI for illicit drug abuse [15].

Despite the potential advantages of initiating treatment in primary care, there continues to be considerable disparity between new knowledge generated by research and clinical practice [8,16]. Although there are a large number of positive research studies and recommendations to routinely carry out screening and BI in primary care, there has been little movement toward adopting it [17–21]. This has been attributed to a lack of effective knowledge transfer to frontline health services. Thus, finding methods to enhance knowledge translation and further understanding of frontline practitioners’ resistance to adopting evidence-based research are key elements in the knowledge translation process [22,23]. Research-based interventions are often perceived as being incompatible with clinical practice because they fail to take the unique culture and context of community services into consideration. Consequently, it has been suggested that researchers go beyond asking questions about the ‘efficacy’ of interventions under ideal circumstances, to questions of ‘will it work?’ or ‘is it worth it?,’ in specific local circumstances [24].

### Quebec, Canada-a unique opportunity to study the implementation of frontline services for substance dependence

The re-structuring of healthcare services in Quebec, Canada, instituted by the Ministry of Health and Social Services of Quebec (MSSS) offered a new and particularly unique opportunity to study the implementation of frontline services for substance dependence. The MSSS recently issued provincial policies for addictions prevention and early intervention titled ‘Plan d’action interministériel en toxicomanie 2006–2011’ (Action Plan on Addiction) and ‘Offre de service-Orientations relatives aux standards d’accès, de continuité, de qualité, d’efficacité et d’efficience: Programme-Services dépendance, 2007–2012’ (Addictions Program and Services) [25,26]. These policies provide examples of a system-wide effort to drive changes in frontline healthcare practices. The initial ‘Action Plan on Addiction’ outlined a collaborative effort of the MSSS and other governmental ministries to address prevention and early intervention for substance use disorders. The follow-up policy document ‘Addictions Program and Services’ mandated province-wide integration, coordination and continuity of care to be provided by frontline Health and Social Services Centres called Centre de santé et de services sociaux (CSSS) and second line addictions treatment centres (CRPAT), promoting a population-based approach to prevention, screening, early intervention and treatment of substance problems in Quebec.

The CSSS are responsible for overseeing the primary health, mental health and social services needs of specific geographical regions in Quebec [27]. In the policy documents described above, the CSSS were mandated to provide services for a) screening^b^ for alcohol, drug and gambling problems and orientation to appropriate services; b) early intervention; c) psychosocial follow-up during specialized treatment; d) methadone maintenance with psychosocial and medical follow-up; and e) detoxification with psychosocial follow-up. It is notable that while the rationale for the program and the services to be developed were outlined within the Action Plan on Addiction and the Addictions Program and Services policy documents [25,26], the methods of implementation were not specified. Thus, each CSSS was responsible for developing an addictions program that best fit their regions’ needs, and developing their own implementation methods and time lines. It is within the context of the MSSS Action Plan on Addiction that the current research protocol was developed. Phase 1 of the protocol was designed to test specific implementation methods based upon principles of integrated knowledge translation and participatory action research [28–31].

Phase 2 of the protocol was designed to examine factors influencing wide-scale implementation of frontline services for substance dependence in the province of Quebec. In this context, it is notable that to date there has been no examination of the impact of the MSSS policies on the development of frontline addictions services in Quebec.

### Conceptual framework

The research program was designed by a team of researchers, clinicians and administrators with broad expertise in addictions, primary care, mental health, family medicine, participatory action research, and knowledge translation research. The partnership included the Addictions Unit of the McGill University Health Centre (MUHC), the CSSS de la Montagne and Participatory Research at McGill (PRAM). Throughout the research process, the Team will utilize an integrated knowledge translation (iKT) strategy to build collaborative mechanisms for knowledge exchange between researchers, addiction specialists and practitioners [29,30]. Incorporation of knowledge has been described as a transforming process (e.g., adding new experiences and information to existing knowledge, dialogue with colleagues, identifying beliefs and feelings about new information) rather than a linear transfer of information into practice [32]. Successful iKT has been described as an interactive process involving a bi-directional flow of knowledge between researchers and users during all stages of protocol development in a collaborative process [28,29,33–35].

The research will directly examine the process of knowledge uptake and barriers to transfer using both qualitative and quantitative methodologies. Evaluation will involve multiple measures, time points and domains, taken from the perspective of multiple stakeholders. Thus, research in ‘real-world’ clinical settings will be conducted and extensive evaluation mechanisms will be put in place in to assess the outcomes and impact of the treatment program, as well as the networking, team-building, and knowledge transfer activities.

The knowledge translation strategies adopted in Phase 1 of the research were also guided by the conceptual framework outlined in the Ottawa Model for Research Use [36]. The Ottawa Model was developed to help guide the transfer of research innovations into practice, and it is an example of a planned change theory aimed at affecting change at the organizational level [37]. The Ottawa Model assesses key elements related to the innovation, the potential adopters, and the practice environment for barriers and facilitators that could influence adoption. Knowledge translation strategies are selected and monitored, followed by on-going monitoring of the innovation’s adoption throughout the organization, and finally an evaluation of the outcomes or impact of the innovation. The distinct steps in this conceptual framework helped to guide the research team throughout Phase 1.

### Objectives and specific aims

Phase 1 will utilize an iKT strategy to build collaborative mechanisms for knowledge exchange between researchers, addiction specialists and frontline practitioners, and directly examine the process of knowledge uptake and barriers to transfer using both qualitative and quantitative methodologies. Phase 2 will explore the process of policy dissemination, and the level of adoption and implementation of frontline services for substance dependence throughout the province of Quebec.

#### Specific aims:

1. To study the effectiveness of the integrated knowledge transfer and program implementation process. Evaluation will involve multiple domains (attitudes, readiness for change, training needs, knowledge, confidence in clinical skills, satisfaction with training and the iKT process) taken from the perspective of multiple stakeholders using a variety of methods including questionnaires, focus groups, and in-depth key informant interviews. Measures will be taken to monitor utilization of the program components by frontline staff. Evaluation will be conducted over the six-month baseline period prior to training and program implementation (Time 1), immediately after training and program implementation (Time 2), and at the one-year (Time 3) follow-up time points (Phase 1).
2. To measure changes in service provision following program implementation using chart reviews as well as administrative databases provided by the provincial health database RAMQ (Régie de l’assurance maladie du Québec) (Phase 1). Service delivery will be measured using a pre-post design in which the numbers of patients screened, diagnosed and treated will be monitored over a six-month baseline period prior to training and program implementation (Time 1), as well as at Times 2 and 3 (Phase 1).
3. To determine the barriers and facilitators associated with implementation of frontline services for substance dependence in Quebec (Phase 1 and Phase 2).
4. To determine the level of program development and service implementation across the province of Quebec based on government policies in addiction services (Phase 2).

## Methods

Phase 1 is a pre-post mixed-methods design, with observations at multiple points in time, with one case intervention CSSS and one comparison CSSS site. The intervention CSSS consists of three local health clinics located in Montreal. Participants will be frontline healthcare providers (social workers, nurses, and master’s level counsellors and other allied health professionals), and management from the community health teams at the CSSS working in adult and youth mental health, teams for youth and families at risk, and other general psychosocial health services. Staff directories will be provided by the organizations, and the identified teams will be invited to participate in the research by the Team research coordinator.

## Intervention

### Training program

The training program titled ‘Treating Substance Dependence and Mental Illness: Tools for the Front Line Practitioner’ was designed by addictions specialists from the Addictions Unit of the MUHC to facilitate the adoption of evidence-based practices by frontline practitioners. The intended end-users are the physicians, nurses, and other health professionals within primary healthcare clinics who are likely to encounter drug or alcohol abusers in their daily practice. This comprehensive training program for screening and brief interventions will be used as a starting point to be tailored and implemented based on the participants’ reported needs. The program consists of clinical guidelines and best practices for treatment, as well as training modules that make use of case studies, videotaped interviews, role-play using actors, interactive lectures and workshops. Modules include materials related to screening, assessment, diagnostic criteria, pharmacotherapy, brief intervention (using a validated five-session abstinence-oriented brief intervention program developed and tested at the Addictions Unit), as well as the identification and treatment of co-morbid mental illness.

### Transfer method

As part of the iKT approach, the Team chose an implementation method that will involve a collaborative approach through the insertion of an experienced, certified addictions specialist (Addictions Program Coordinator) into the frontline community health teams involved in the project. During the preparation phase, the Addictions Program Coordinator will be hired and complete further specialized training in the principles and practices of iKT [28]. The Team objectives for the training are to provide tailored comprehensive evidence-based training to health workers in order to increase participants’ knowledge, confidence and skills in substance dependence and co-morbid mental health, and to increase participants’ use of screening and brief interventions to better address the needs of clients experiencing substance dependence and co-morbid mental health problems.

Over an 18-month training and implementation period, the APC will a) act as a liaison among team members (university-based addiction specialists, administrators, frontline staff); b) evaluate the current conditions of practice at each clinic; c) arrange for training of designated frontline staff on all the treatment program modules; and d) implement the treatment program in all clinics, in collaboration with the health professionals and administrators of the CSSS. Implementation in this context will involve the development of treatment protocols and procedures that are tailored to each clinic, and developed jointly with the practitioners on-site. Note that the APC will not directly provide clinical care during the implementation period, and that examination of the process of implementation and measurement of program effectiveness (outcomes for patients, changes in healthcare service delivery) will be conducted by a separate, independent research team consisting of a Clinical Research Coordinator and Research Assistants. A Training Committee will be developed in order to collaboratively plan the knowledge translation and training activities at the local community service centres. Decisions regarding who and how many to train, and their expected role will be made by the CSSS management team. A second CSSS will be used as the control site, receiving no intervention.

### Phase 1 data collection & analysis

#### Interviews & focus groups

The semi-structured interview guide will explore frontline practitioners’ and managers’ current knowledge, attitudes, confidence and practices related to substance dependence, in addition to their perceived training needs and opinions regarding potential barriers for implementing services for screening and brief intervention. Interviews and focus groups will be audio recorded, transcribed verbatim, and subsequently verified by research assistants and the research coordinator to ensure accuracy and comprehensiveness. Interviews will take place at multiple time points: pre- and post- training and implantation of the APC. Qualitative analysis will be conducted by experienced data analysts guided by the interpretive description design [38] and the framework analysis method [39], which involves inductive or emergent coding, while still allowing for the inclusion of a priori coding. A five-step process was followed including: familiarization with the data, identifying a thematic framework, indexing of themes, charting, mapping, and interpretation. Qualitative analysis software, QSR Nvivo 9 (QSR International) will be used to aid in the systematic indexing, coding and organization of the data. Analysis of qualitative data will identify social, cultural and organizational factors affecting the uptake of practice recommendations among practitioners. Coding and analysis will be conducted independently by several analysts in order to establish consensus of the main themes. Qualitative and quantitative data from the APC’s process recording will also be used to add depth to the qualitative analysis, triangulate the interview and questionnaire data and further identification of the perceived barriers to program implementation. Systemic, structural and staff-level barriers will be evaluated. Regular partnership meetings with the CSSSs will allow for further discussion and interpretation by all the research team members.

### Questionnaires

#### Evidence-based practice attitude scale (EBPAS)

The EBPAS is a well validated tool that was developed to assess mental health service providers’ willingness to adopt an innovation given their attitudes toward the use of evidence-based practices (EBP) [40,41]. This 15-item questionnaire has four subscales: Appeal, Requirements, Openness and Divergence. This questionnaire is widely used in implementation research; it has been well validated [40,42] and shown to be reliable and generalizable [41]. Statistical analysis of all questionnaires will be completed using SPSS Version 21 (IBM Ltd).

#### Organizational readiness for change (ORC)

The organizational readiness for change (ORC) assessment focuses on organizational traits that predict program change, and it was specifically designed in relation to substance dependence [43]. The ORC measures the impact of organisational functioning on knowledge transfer using a 115-item Likert-type scale with items grouped conceptually in four areas-motivation for change, resources, staff attributes, and organisational climate-across 18 scales [43,44]. The ORC has been validated [43], and shown to have good predictive validity [45]. The characteristics of an organisation’s climate that facilitate change [46] and the concept of readiness for change [47] are issues central to the adoption and routine use of innovative practices in mental health and community health services. Overall, the ORC was selected for use as it can contribute to the study of organizational change by identifying the functional barriers involved [43].

### Socio-demographic questionnaire

A socio-demographic questionnaire assessed basic demographics including age, sex and education for all participants. Additional questions were included in order to assess professional experience working in mental health and addiction services, and training specific to substance use disorders.

### Patient chart review & service provision data

One of the objectives of the Phase 1 data collection will be to measure changes in service provision at each clinic site. Data will be collected prior to training and program implementation (Time 1); immediately after training and program implementation (Time 2), and at the one-year (Time 3) follow-up time points.

Outcome measures for this objective will be pre-post measures of the number and percentage of presenting patients screened and assessed for addiction and co-morbid mental health problems at each of the clinics, the number and percentage of the clinic population at each clinic identified with substance use disorders with or without co-morbid mental illness, the number, percentage and type of different interventions completed (including screening, assessment, detoxification, psychiatric evaluation, and BI), and the number of referrals to secondary specialized services. In addition, information will be gathered on total health service utilization (number and length of different interventions, number of sessions and number of different practitioners involved in the management of each case). This information will be gathered from patient charts, as well as administrative databases obtained from the provincial health services (RAMQ).

### Phase 2 summary

Phase 2 is conceptually informed by the early stages of Phase 1 and will include data gathering from additional CSSS throughout the province of Quebec. It builds on the qualitative component of the initial design, replicating some of its original aims and expanding its scope in order to allow for conceptual continuity and for analytical generalizability. Semi-structured interviews with managers and staff at each CSSS will be conducted in order to evaluate how frontline services for addiction and substance dependence have developed in various parts of the province. The interviews with managers and staff will document service provision as well as organizational policies and practices. More broadly, Phase 2 will investigate the implementation of a government policy in primary healthcare settings, identifying the factors acting as facilitators and barriers to the adoption of government initiatives at the community health services level in the province of Quebec.

### Phase 2 participants

The Phase 2 sites will be selected from among the 94 CSSSs in the province of Quebec. An initial letter of invitation to participate in the research study will be sent out to all CSSSs in Quebec in order to determine interested organizations. Given the need for local ethics involvement, it is important to ascertain interest in participating prior to site selection and ethics procedures. A purposeful sampling strategy will be used to select 21 interested CSSSs in order to explore the services and implementation process within diverse contexts and geographical regions.

A maximum of four key informants will be recruited at each of the selected CSSSs for a maximum of 84 participants. The interviews will be conducted with key informants whose professional position within the CSSS is closely related to the Addictions Programme-services development and implementation. Following a logic of purposive stakeholder sampling [48], where the major stakeholders are identified and selected based on potential involvement in the decision-making processes related to the Addictions program within their CSSS. The positions selected for recruitment depend on the organizational layout of each site but are similar to: a) Director of Services (e.g., general services, specific services, mental health services, public health, or community health), b) Coordinator of services (addictions services, mental health services, public health, or community health), c) Program Manager (for addictions services or mental health services), and d) Frontline healthcare professional designated to work in addictions. Potential participants will be contacted and invited to participate once ethics approval has been obtained.

### Phase 2 data collection & analysis

Data for the Phase 2 objectives will be collected through semi-structured interviews (with supplemental completion of a checklist of current frontline services for addictions); interviews will be approximately 1.5 hours in duration. Interviews will be audio recorded and transcribed verbatim by a research assistant. Transcriptions will be subsequently reviewed by another research assistant to ensure accuracy and comprehensiveness. Nvivo 9 (QSR International) qualitative data analysis software will be used to manage the qualitative data. The coding strategy will be based on variables associated with the frontline services outlined in the addiction program and services as well as themes emerging over the course of the project. Analysis will be completed through a process of familiarization with the data through multiple readings of the transcripts, identification of major themes and line-by-line coding, and guided by the framework analysis method, similar to Phase 1 [39]. Data will be sorted by case (CSSS) as the main unit of analysis to allow an in-depth exploration of each CSSS and a comparison of cases. Data from the different case participants will be triangulated to increase the validity of the CSSS case descriptions, and when available, case documentation such as organizational guidelines and strategic plans will be reviewed and integrated for a more thorough understanding of the process and level of implementation of frontline addiction services at each site.

### Ethical approval

Phase 1 study procedures have been approved by both the MUHC Research Ethics Board and the Research Ethics Committee of the partner CSSS, and endorsed by the local ethics committee from the comparison CSSS. For Phase 2, as per the provincial multicentre ethics procedure, one of the CSSS’s Research Ethics Committees is acting as the primary ethics committee and has approved this study. The 20 additional local ethics authorities have endorsed this decision, as well as the MUHC Research Ethics Board. All participants in both phases of the study will sign a written consent form. The confidentiality of participants will be protected with coded processing of data.

### Study status

The study will be completed in March 2016. At the time of submission, the Team has completed three data collection time frames for Phase 1. Phase 2 received ethical approval in June 2013, and recruitment and data collection are ongoing.

## Discussion

The province of Quebec, Canada, aims to improve the organization and delivery of services for substance use disorders through the implementation of frontline practices and improved continuity of care. This study will offer a comprehensive understanding of the barriers and facilitators impacting effective implementation of frontline services for problematic substance use. Study strengths include the collaborative iKT approach involving frontline health professionals, healthcare managers, physicians and experts in participatory research, which is central to Phase 1. This allows all partners to collaborate in a shared approach to the challenges related to the implementation of evidence-based services for addictions at the community level. The Team has expertise in addictions and co-morbid mental health, knowledge translation, knowledge-user engagement, and health services program development and implementation.

Phase 2 is an innovative approach to exploring the barriers to mandated implementation of frontline services for addictions. This will be the first study exploring the wide-scale implementation of policy related to substance dependence in the province of Quebec, and it should yield needed information about how to effectively implement policies into clinical practice.

Some limitations of the study include possible recruitment bias, as the CSSSs that agree to participate may be considerably different from those who are unwilling to participate (e.g., CSSSs with low levels of implementation may not want to participate in the study). To address this bias, recruitment efforts clearly indicated the desire for both low and high level implementation, and efforts have been made to recruit sites with a range of services. Given that this research is conducted in the CSSS and their local community services centres in Quebec, Canada, the transferability of findings to other frontline healthcare settings may not be applicable. The Team attempted to increase generalizability of findings for the Quebec healthcare system by including organizations from 13 different healthcare regions within the province. Lastly, the qualitative data collected will rely on self-report of the current practices and services. Given the multiple clinical settings with various administrative systems, it was not feasible for this study to collect quantitative data of clinical services provided at each site. This type of information would provide valuable supplementary data on the level and efficacy of services implemented and could be a potential direction for future research.

The CSSS in Quebec, Canada, have been mandated to make significant efforts to implement frontline services for screening and early intervention for substance use disorders. Although the adoption of EBP for substance use problems is an identified priority in Quebec’s healthcare policies, there are extensive implementation challenges. Without further understanding of the real-time challenges (and possible need for additional and sustained implementation support), the potential public health benefit of wide-scale screening and early intervention may not be realized. This study has the potential to impact public health by developing a better understanding of the local barriers and facilitators to improving implementation of effective practices addressing addictions in frontline health services.

## Endnotes

^a^Substances refer to all categories of licit and illicit psychotropic drugs including alcohol, cannabis, benzodiazepines, dissociative anaesthetics, psychostimulants (cocaine, amphetamines), opiates (heroin, pain killers), etc.

^b^The MSSS does not use the term screening (‘dépistage’) in relation to substance abuse and addictions but reserves that term for purely ‘physical health’ screening. The MSSS uses two terms, ‘repérage’ and ‘détection’: repérage is the systematic screening of all clients requesting care at the CSSS, while detection is the assessment conducted following a positive screening during repérage [25,26].

## Abbreviations

APC: Addictions Program Coordinator
BI: Brief interventions
CIHR: Canadian Institutes of Health Research
CRPAT: Second line addictions treatment centres
CSSS: Centre de Santé et de Services Sociaux, Health and Social Services Centres
EBP: Evidence-based practice
EBPAS: Evidence-Based Practice Attitude Scale
iKT: Integrated knowledge translation
MSSS: Ministry of Health and Social Services of Quebec
MUHC: McGill University Health Centre
ORC: Organizational Readiness for Change
PRAM: Participatory Research at McGill
RAMQ: Régie de l’assurance maladie du Québec
